# Plasma cells within granulomatous inflammation display signs pointing to autoreactivity and destruction in granulomatosis with polyangiitis

**DOI:** 10.1186/ar4490

**Published:** 2014-02-20

**Authors:** Antje Mueller, Christoph Brieske, Susanne Schinke, Elena Csernok, Wolfgang L Gross, Katrin Hasselbacher, Jan Voswinkel, Konstanze Holl-Ulrich

**Affiliations:** 1 Department of Rheumatology, University of Luebeck, Luebeck, Germany; 2 Department of Rheumatology & Clinical Immunology, Medical Center Bad Bramstedt, Bad Bramstedt, Germany; 3 Department of Otorhinolaryngology, University of Luebeck, Luebeck, Germany; 4 Department of Hematology, University Hospital Saint Antoine & University Pierre et Marie Curie, Paris, France; 5 Institute of Pathology, University of Luebeck, Luebeck, Germany

## Abstract

**Introduction:**

Plasma cells residing in inflamed tissues produce antibodies in chronic inflammatory and systemic autoimmune diseases. This study examined if plasma cells, located within inflamed nasal tissue in granulomatosis with polyangiitis (GPA), express features potentially associated with the autoimmune and destructive character of this disease.

**Methods:**

*Ig* gene mutation patterns of individual tissue-derived plasma cells from GPA (*n* = 5) were analyzed, by using laser-assisted microdissection followed by semi-nested polymerase chain reaction (PCR). Signs of B-lymphocyte maturation (ectopic lymphoid structures, ELS) and survival (a proliferation-inducing ligand, APRIL; B-cell maturation antigen, BCMA; transmembrane-activator and calcium modulator and cyclophilin interactor, TACI; receptor activator of nuclear factor κB ligand, RANKL) were examined in nasal tissues or serum, respectively, by using immunohistochemistry/fluorescence and enzyme-linked immunosorbent assay, ELISA.

**Results:**

Plasma-cell derived *Ig* genes (light- and heavy-chain pairs, *n* = 4; heavy chains, *n* = 33) resembled mutation patterns seen in other autoimmune diseases, predominantly displaying selection against replacement mutations within the framework region of *Ig* genes (10 of 15), which is responsible for structural integrity. Ectopic lymphoid structures were similar between GPA and a disease control (that is, unspecific chronic rhinosinusitis. However, histomorphologic features distinguishing GPA from rhinosinusitis (that is, neutrophilic microabscess and granuloma) expressed considerable amounts of membrane-associated and secreted APRIL, respectively. The latter was co-localized with CD138 and found in close proximity to cells expressing IgG, TACI, and BCMA. Interestingly, plasma cells strongly expressed receptor activator of nuclear factor κB ligand (RANKL), apart from fibroblast-like cells.

**Conclusions:**

Plasma cells within granulomatous inflammation appear to display features that might be required for autoreactivity and, possibly, RANKL-mediated destruction in GPA.

## Introduction

Granulomatosis with polyangiitis (GPA/Wegener) is a multisystem disease of unknown etiology, characterized by granulomatous manifestations in the respiratory tract and systemic necrotizing vasculitis. Anti-neutrophilic cytoplasmic antibodies (ANCA) with specificity for proteinase 3 are a defining feature of this disease, but other autoantibodies are found as well [1,2]. Clinical symptoms are often due to necrotizing granulomatous inflammation, predominantly in the respiratory tract, leading to fibroblast-mediated cartilage/bone destruction and to vasculitis, probably autoantibody mediated [1,3,4]. Inflamed tissue within nasal mucosa displays the pathognomonic triad consisting of ill-defined granuloma, geographic necrosis, and vasculitis [5], accompanied by prominent neutrophil infiltration (microabscess) and lymphoplasmocytic aggregates [3,5,6].

Recently, we detected mutated Ig variable (V) region genes in nasal tissue in GPA, and some of the CD20^+^ B cells produced autoantibodies [7]. Thus, we assumed that autoreactivity develops in inflamed nasal tissue, probably via ectopic lymphoid structures (ELS). ELS are considered the morphologic basis of B-cell autoimmunity in rheumatoid arthritis (RA) [8]; however, this association was questioned [9]. Further, B cells can be depleted via anti-CD20 therapy, inducing remission in GPA [10]. Nonetheless, relapses occur, suggesting that plasma cells, surviving in niches and producing autoantibodies [11], could be responsible. Cells expressing B cell-activating factor, B cell-activating factor receptor, and a proliferation-inducing ligand (APRIL) were shown in GPA mucosa [12], promoting the niche concept. To search for alterations, plasma cells derived from inflamed nasal tissue in GPA were analyzed in terms of mutation pattern of their *Ig* genes and compared with controls [13], after laser-assisted microdissection and semi-nested PCR.

To investigate a relevance for B-cell autoimmunity in GPA, ELS were examined and compared with a non-autoimmune disease control, by using immunohistochemistry. Because plasma cell survival is mediated through APRIL signaling via B-cell maturation antigen (BCMA) or transmembrane-activator and calcium modulator and cyclophilin ligand interactor (TACI) [14], their expressions were investigated, by using immunohistochemistry/-fluorescence and Elisa. Because APRIL binding to BCMA led to elevated receptor activator of nuclear factor κB ligand (RANKL) levels [15], its tissue expression was evaluated as well.

Our results indicate altered Ig V gene-mutation patterns in plasma cells residing in inflamed nasal tissue. The presence of ELS in GPA suggests the possibility of a role in developing autoreactive B cells [7]; however, the phenotypical properties of ELS did not differ from a non-autoimmune inflammatory disorder (that is, chronic rhinosinusitis (CRS)). In contrast, plasma cell survival seems to be supported by distinct histomorphologic structures in GPA (that is, neutrophilic microabscess and granuloma), expressing the survival factor APRIL. Co-localization of APRIL and CD138 allows recognition by the receptor TACI. RANKL expression by cells with a plasma cell-like appearance might serve as an indication of binding between APRIL and the receptor BCMA.

## Methods

### Patients and tissues

Sinunasal biopsies were taken from 26 GPA patients, 20 patients with unspecific CRS, and one patient each with rheumatoid arthritis (RA) and sarcoidosis. Patients’ written consent according to the Declaration of Helsinki was obtained, and the study design was approved by the ethics committee of the University of Luebeck (07–058). Patient characteristics are summarized (Additional file 1: Table S1). Formalin-fixed and paraffin-embedded nasal and lung biopsies of 22 GPA patients were selected for immunostaining (Additional file 1: Table S2), and freshly frozen nasal biopsies of five GPA patients (proteinase 3-ANCA^+^) were chosen for *Ig* gene analysis. Markers of the histomorphologic triad of GPA were not present in these five biopsies, but lymphoplasmocytic infiltrates, and three patients had a history of GPA-related histology.

### Isolation and characterization of plasma-cell-derived Ig V region genes

This was conducted as described before [6], with the exception of staining with anti-CD138 (MI15; Dako, Hamburg, Germany) followed by anti-mouse HRP-conjugate (Zytomed Systems, Berlin, Germany) and aminoethylcarbazole (Dako). PCR products were identified by using IMGT/V-Quest [16]. All sequences have been submitted to GenBank (accession numbers: JN990775-JN990790, JN990797-JN990808, JQ240200-JQ240203, JQ693385-JQ693389, JQ715619-JQ715622).

### Immunohistochemistry

The following primary mouse, rabbit, or rat antibodies were used: anti-CD3 (Dako), anti-CD4 (4B12; Biogenex, Fremont, CA, USA), anti-CD8 (BC/1A5; Biocare Medical, Concord, MA, USA), anti-CD20 (L26), anti-CD21 (1 F8), anti-CD35 (Ber-MAC-DRC), anti-CD57 (TB01), anti-CD68 (PGM1), (all Dako), anti-CD23 (SP23; Biomol, Hamburg, Germany), anti-CD38 (38C03; Labvision, Dreieich, Germany), anti-peripheral node addressin (PNAd: MECA-79; BD, Heidelberg, Germany), anti-APRIL (Stalk-1/ED; MyBioSource, San Diego, CA, USA; Ap2; Enzo Life Sciences, Lörrach, Germany), anti-RANKL (MIH24; Biolegend, San Diego, CA, USA), anti-TACI (R&D Systems, Wiesbaden, Germany), and anti-BCMA (Sigma, München, Germany). Neutrophils were detected enzymatically by using naphthol ASD chloroacetate [17]. After deparaffinization and rehydration of sections (3 to 4 μm), antigens were retrieved by heating in pH 6.0 or pH 9.0 buffer. Nonspecific binding was minimized by using protein block (Dako). To provide for controls, sections were stained with appropriate isotype controls (Dako; AbD Serotec, Düsseldorf, Germany) instead of primary antibodies. Stainings for CD marker, PNAd, APRIL, TACI, and RANKL were carried out by applying a peroxidase-based protocol [6]. CXCL13 and CCL21 were stained with goat anti-CXCL13 and goat anti-CCL21 (R&D Systems), respectively, by using an avidin/biotinylated enzyme complex-based protocol [4]. Double stainings for secreted APRIL and goat anti-human IgG F(abʹ)_2_ fragment (Dianova, Hamburg, Germany), as well as for CD138 and BCMA, were performed by using the Envision Doublestain kit (Dako) according to the manufacturer’s instructions, with the exception of using donkey anti-goat-HRP-conjugated antibody (Dianova) for IgG detection. Images were acquired by using a slide scanner and corresponding software (Mirax Midi; Zeiss, Göttingen, Germany).

### Double immunofluorescence staining

In brief, sections were stained for CD138 followed by donkey anti-mouse AF488 Fab fragment (Dianova). Thereafter, secreted APRIL was stained by using Ap2 antibody followed by goat anti-mouse AF568 IgG. Nuclear staining was done by using 4′,6-diamidin-2-phenylindol (DAPI) followed by mounting with ProLong Gold reagent (all Life Technologies, Karlsruhe, Germany). Sections were viewed on a laser-scanning microscope IX81 and confocal stacks modeled with the FV10-ASW v03 software (Olympus, Hamburg, Germany).

### ELISA

APRIL was measured in sera of GPA patients (*n* = 20), CRS patients (*n* = 10), and healthy controls (*n* = 13) by using an ELISA according to the manufacturer’s instructions (eBioscience, Frankfurt/M, Germany).

## Results

### Altered mutation pattern of plasma cell-derived Ig genes in nasal tissue in GPA

Of 41 productively rearranged Ig V region gene fragments, representing 37 dispersely distributed CD138^+^ plasma cells, 15 (37%) yielded significantly mutated Ig genes compared with corresponding germline genes (Table 1; Additional file 1: Table S3). Selection was calculated based on replacement to silent mutation ratios in a complementarity-determining region (CDR) and framework region (FR), according to [18]. Regarding the FR, which determines the shape of the B-cell receptor, none of the 15 genes exhibited selection for replacement mutations, whereas selection against replacement mutations was detected in 10 genes. Mean mutation frequencies of L- and H-chain genes were 3% and 8%, respectively. Although statistically insignificant (probably due to the low number), VH genes showed overrepresentation of VH1 versus underrepresentation of VH4 rearrangements and a prolonged CDR3 (Additional file 1: Figure S1).

**Table 1:** **Overview of selection (*P*** **values) against or for replacement mutations within framework region (FR) and CDR of plasma cell-derived Ig genes in nasal tissue in GPA**^**a**^

**V region subfamily**	**FR negative**	**CDR negative**	**CDR positive**
VH1-3	*P* ≤ 0.002^b^	*P* ≤ 0.03^b^	
VH1-46			*P* < 0.02
VH1-69	*P* ≤ 0.01(2),^e^*P* ≤ 0.02 (2),^f^*P* < -0.05		*P* < 0.01
VH3-23	*P* ≤ 0.02^c^	*P* ≤ 0.01^c^	
VH3-30	*P* ≤ 0.001		
VH3-74	*P* ≤ 0.02		
VH4-39			*P* < 0.02
VH4-4	*P* ≤ 0.01^d^	*P* ≤ 0.03^d^	
Vκ1-39			*P* < 0.03
Vκ2-28			*P* < 0.04

^a^The focused binomial test [18] was used to estimate selection, yielding *P* values with statistical significance at <0.05.

^b,c,d^These three VH genes showed negative selection in both FR and CDR.

^e,f^Two and two individual VH genes each, respectively, displayed identical *P* values.

### Ectopic lymphoid structures are similar between GPA and disease control

**Figure 1:**
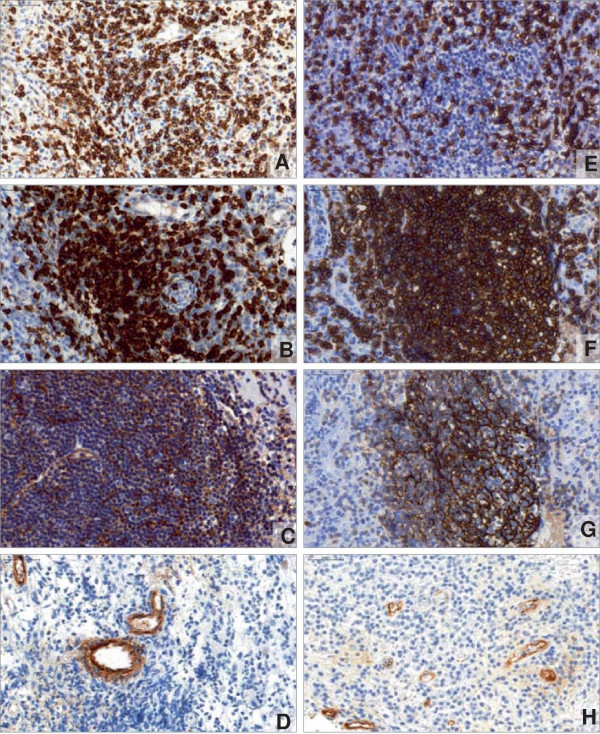
**ELSs in nasal tissues do not differ between GPA and CRS. (A through D)** Immunohistochemistry of GPA displaying CD3^+^ T lymphocytes (**A**, brown) surrounding a primary CD20^+^ B-cell follicle (**B**, brown color) with a CD21^+^ FDC network (**C**, brown), which could be located in proximity to PNAd^+^ high endothelial venules (**D**, brown). **(E through H)** Immunohistochemistry of CRS showing CD3^+^ T lymphocytes (**E**, brown) surrounding a primary CD20^+^ B-cell follicle (**F**, brown) with a CD21^+^ FDC network (**G**, brown) that could be located near PNAd^+^ high endothelial venules (**H**, brown). Nuclei are stained in blue with hematoxylin (Figure 1A-H, Figure 2A-G, K, L).

ELSs were frequently present within granulomatous inflammation, ranging from aggregates to highly organized forms. Nonetheless, this was also true for the disease control, unspecific CRS (Figure 1A-H; Additional file 1: Table S4). Almost all tissues exhibited lymphocytic aggregates resembling follicular structures, most of them characterized by networks of CD21^+^ follicular dendritic cells (FDCs). High endothelial venules expressing PNAd were found in the majority of tissues, often located close to follicular structures. Cells staining positive for the lymphoid chemokine CXCL13 [8] were indicative of FDCs, but networks could not be detected (Additional file 1: Figure S2A-B). Although vessels expressing the lymphoid chemokine CCL21 were observed (Additional file 1: Figure S2C-D), perivascular CCL21^+^ stromal cells, being described as important for ELSs in RA [19], could not be found. Plasma cell infiltrates were seen in nearly all tissues.

### Neutrophils, macrophages, and giant cells forming granulomatous inflammation are major sources of APRIL

**Figure 2:**
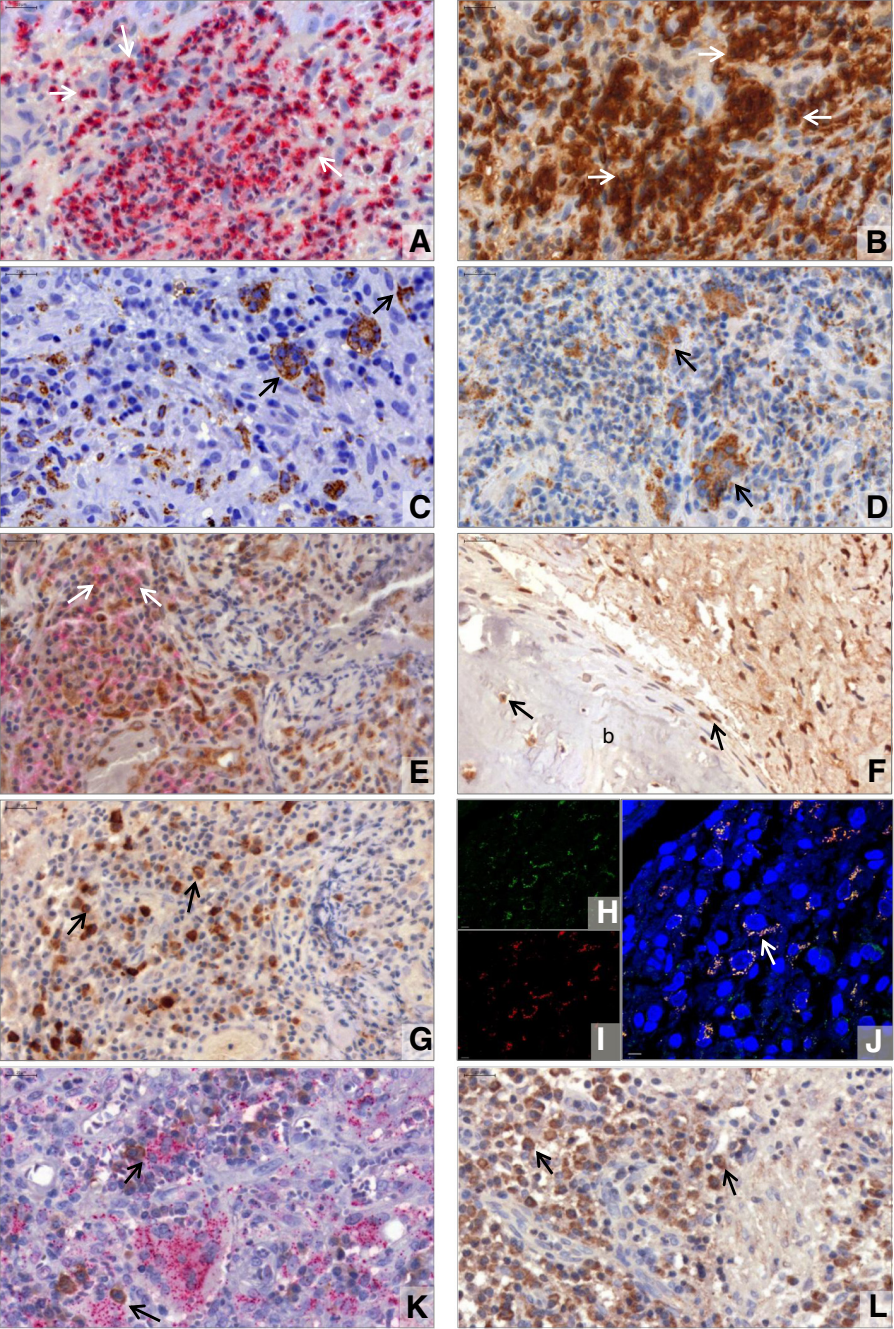
**Granulomatous inflammation supports plasma cell survival in GPA. (A through D)** Immunohistochemistry displaying neutrophils (**A**, red arrows) forming a microabscess and strong expression of membrane-associated APRIL (**B**, brown arrows) in the area corresponding to the neutrophilic microabscess. Immunohistochemistry showing macrophages and giant cells forming a granuloma (**C**, brown) and strong expression of secreted APRIL (**D**, brown arrows) by macrophages and giant cells. **(E through G)** Double immunohistochemistry displaying BCMA (**E**, brown) in close vicinity to CD138 (**E**, red). Cells positive for BCMA and CD138 are marked (arrows). Immunohistochemistry indicating different types of RANKL-expressing cells, close to bone **(b)** (**F**, brown arrows) and with a plasma cell-like morphology (**G**, brown arrows). **(H through J)** Immunofluorescence staining demonstrating that CD138 (**H**, green) is co-localized with secreted APRIL (**I**, red color) on plasma cells (**J**, merger of **H** + **I**, arrow). Nuclei are stained in blue with 4′,6-diamidin-2-phenylindol (DAPI). Scale bar, 5 μm. (**K**, Double immunohistochemistry depicting that secreted APRIL (red) is expressed in direct vicinity of IgG^+^ cells (brown arrows). **(L)** Immunohistochemistry showing that TACI^+^ cells (brown arrows) exhibit a plasma cell-like appearance.

Our results demonstrated that PMNs, accumulating in microabscesses, are a major source of membrane-associated APRIL in GPA (Figure 2A,B; Additional file 1: Table S5). Diffusely distributed neutrophils expressing membrane-associated APRIL were also present in CRS (Additional file 1: Figure S3A). Granulomas, composed of macrophages and giant cells, displayed substantial expression of secreted APRIL (Figure 2C,D; Additional file 1: Table S3). Because tissue-infiltrating neutrophils were negative for secreted APRIL, we assumed that after furin cleavage, APRIL is secreted and taken up by other cells, a mechanism that has been suggested before [20]. Further, epithelial cells expressed secreted APRIL in GPA and CRS (Additional file 1: Figure S3B). Increased amounts of APRIL were also secreted in peripheral blood in GPA when compared with CRS and HC (Additional file 1: Figure S4).

### APRIL receptors (BCMA, TACI) and RANKL are expressed by plasma cells

BCMA was expressed by plasma cells (Figure 2E), lymphocytic and fibroblast-like cells. The latter also stained positive for RANKL, but unexpectedly, a substantial RANKL expression was found in cells exhibiting a plasma cell-like morphology (Figure 2F,G). The receptor TACI recognizes multi-/oligomerized APRIL cross-linked via heparan sulfate proteoglycans such as syndecans [14]. Syndecan-1 (CD138) and secreted APRIL were colocalized on different cell types, including plasma cells (Figure 2H through J). Moreover, secreted APRIL was found in the direct vicinity of IgG^+^ cells (Figure 2K). Thus, clustered APRIL could interact with TACI, which was expressed by cells displaying a plasma-cell-like morphology (Figure 2L). Regarding CRS, cells expressing BCMA, RANKL, or TACI were present (Additional file 1: Figure S5). Additional experiments are warranted to determine whether apparent differences (cell type, numbers) are relevant in comparison with GPA.

## Discussion

Based on previous findings in GPA [7,12] and similarly to other chronic inflammatory diseases [8,20,21], we hypothesize that nonlymphoid tissue, such as the nasal mucosa in GPA, is a place where B/plasma cell-mediated autoreactivity could develop and/or be present. Overall, our results indicate alterations in the *Ig V* genes of tissue-derived plasma cells and histomorphologic support for plasma cell development and survival in nasal tissue in GPA. In more detail and regarding genetic features of plasma cells, we observed similarities between our current and previous [7] results, a meta-analysis of other studies in various autoimmune diseases [13], and findings in rheumatoid synovium [22]. Despite these similarities (showing, for instance, a prevalence of selection against mutations in the framework region of *Ig V* genes, which is not seen in controls), we are well aware that altered mutation patterns in the *Ig V* gene regions in themselves are not sufficient to indicate autoreactivity in the granulomatous inflammation. Moreover, aside from the mutation rate, physicochemical effects of mutations leading to amino acid exchanges have to be considered as well [13]. In general, to demonstrate autoreactivity, this must be validated by cloning and expressing the antibodies from the isolated plasma cells and testing them for (auto)antigen specificity, which is part of ongoing work. Apart from a possible association with autoreactivity, mutations in *Ig V* gene regions could be induced by bacterial infections such as *S. aureus*, which has been linked to GPA pathogenesis. Superantigens from *S. aureus*-stimulating T cells have been demonstrated as a trigger for relapses [23]. Nonetheless, it remains to be determined whether B-cell superantigens (for example, staphylococcal protein A, staphylococcal enterotoxin D) [24] might play a role in GPA, which, for example, would lead to alterations in the *Ig V* gene repertoire of B cells.

Besides autoantibody-producing plasma cells, ELS have been implicated in B-cell autoimmunity [8]; however, this is controversially discussed [9]. Although our findings show that ELS are present within granulomatous inflammation and could thus be involved in selection and maturation of B lymphocytes, the results also support the concept that lymphoid structures are a result of chronic inflammation [25]. To understand the role of ELS in GPA in terms of assisting in the development of (auto)immune reactions, more experimental work is needed. Of note, the presence of CD20^+^ cell aggregates and organized ELS in GPA contrasts with another study [12], reporting that B cells mostly are isolated cells. Further, a difference in CCL21 expression between rheumatoid synovium and nasal tissue in GPA with respect to perivascular stromal cells matches a polymorphism in the *CCL21* gene, which is associated with RA but not GPA [26]. Based on our results and corresponding to other studies [9,22], we assume that highly organized ELS are not a specific prerequisite for maturation of (autoreactive) B lymphocytes in GPA. Regarding plasma cell survival and extending previous findings [12] by discriminating between membrane-associated and secreted APRIL [20], GPA-characteristic histomorphology (neutrophilic microabscess, granuloma) seems to concentrate considerable amounts of APRIL, which is comparable with synovium [20], but differs from CRS tissue. The expression of RANKL by fibroblast-like and other cells could be due to an interaction between APRIL and its receptor BCMA, as has been demonstrated for synovial fibroblasts in RA [15]. A contribution of RANKL^+^ plasma cells to destructive or other pathogenic mechanisms in GPA requires further investigation.

## Conclusions

Although tissue-residing plasma cells in GPA display genetic features (indicated by their Ig V region gene-mutation pattern) that are similar to other autoimmune diseases, plasma cell (auto)reactivity in nasal mucosa remains to be demonstrated. A lack of difference between GPA and disease control in terms of ELS phenotype points to less relevance for autoimmune-specific processes. Neutrophils, macrophages, and giant cells forming GPA-characteristic granulomatous inflammation could support survival of plasma cells by providing considerable amounts of both membrane-associated and secreted APRIL. The latter might subsequently interact with the receptors TACI and/or BCMA, expressed by plasma cells. The strong expression of RANKL by plasma cell-like cells, which could be due to binding between APRIL and BCMA, might indicate an unrecognized role for these cells in destructive mechanisms in GPA.

## Abbreviations

ANCA: Antineutrophil cytoplasmic antibody; APRIL: a proliferation-inducing ligand; BCMA: B-cell maturation antigen; CDR: complementarity-determining region; CRS: chronic rhinosinusitis; ELS: ectopic lymphoid structures; FDC: follicular dendritic cells; FR: framework region; GPA: granulomatosis with polyangiitis; PNAd: peripheral node addressin; RA: rheumatoid arthritis; RANKL: receptor activator of nuclear factor κB ligand; TACI: transmembrane-activator and calcium modulator and cyclophilin interactor; V region: variable *Ig* gene region.

## Competing interests

The authors declare that they have no competing interests.

## Authors’ contributions

AM was responsible for conception and design, data collection and analysis, manuscript writing, and final approval of the manuscript. CB participated in data collection and analysis, critical revision, and final approval of the manuscript. SS performed data collection and analysis, critical revision, and final approval of the manuscript. EC aided in conception and design, critical revision, and final approval of the manuscript. WLG contributed conception and design, critical revision, and final approval of the manuscript. KH participated in data collection and analysis, critical revision, and final approval of the manuscript. JV was involved in conception and design, data collection and analysis, manuscript writing, and final approval of the manuscript. KHU aided in conception and design, data collection and analysis, manuscript writing, and final approval of the manuscript. All authors read and approved the final manuscript.

## Supplementary Material

Additional file 1: Table S1 Characteristics of patients at time of biopsy. **Table S2.** Detailed overview about characteristic histomorphologic properties in nasal tissue (*n* = 20) and lung (*n* = 2) of GPA. **Table S3.** Characteristics of *Ig V* genes (*n* = 41) derived from plasma cells (*n* = 37) in nasal tissue (*n* = 5) in GPA. **Table S4.** Summary of histomorphologic features characterizing ectopic lymphoid structures in GPA compared with unspecific CRS. **Table S5**. Summary of APRIL expression by characteristic histomorphologic features in nasal tissue in GPA. **Figure S1.** Sequence analysis of plasma-cell-derived *Ig VH* genes from nasal mucosa in GPA showing the **(A)** number of somatic mutations, **(B)***VH* gene repertoire, and **(C)** length (number of amino acids (aa)) of the CDR3. **Figure S2.** Expression of the homeostatic lymphoid chemokines CXCL13 and CCL21. **Figure S3.** Immunohistochemistry showing subepithelial PMN staining positive for membrane-associated APRIL and epithelium staining positive for secreted APRIL in CRS. **Figure S4.** Increased serum concentration of secreted APRIL in GPA when compared with HC and CRS. **Figure S5.** Immunohistochemistry showing expression of BCMA and D138, RANKL^+^ cells and TACI^+^ cells in CRS. Click here for file
