# Supplementary material for: Plasma cells within granulomatous inflammation display signs pointing to autoreactivity and destruction in granulomatosis with polyangiitis
Source: Arthritis Res Ther. 2014 Feb 20;16(1):R55. doi: 10.1186/ar4490 (PMC3978674; doi:10.1186/ar4490)
Supplement: Additional file 1: Table S1 — Characteristics of patients at time of biopsy. Table S2. Detailed overview about characteristic histomorphologic properties in nasal tissue (n = 20) and lung (n = 2) of GPA. Table S3. Characteristics of Ig V genes (n = 41) derived from plasma cells (n = 37) in nasal tissue (n = 5) in GPA. Table S4. Summary of histomorphologic features characterizing ectopic lymphoid structures in GPA compared with unspecific CRS. Table S5. Summary of APRIL expression by characteristic histomorphologic features in nasal tissue in GPA. Figure S1. Sequence analysis of plasma-cell-derived Ig VH genes from nasal mucosa in GPA showing the (A) number of somatic mutations, (B)VH gene repertoire, and (C) length (number of amino acids (aa)) of the CDR3. Figure S2. Expression of the homeostatic lymphoid chemokines CXCL13 and CCL21. Figure S3. Immunohistochemistry showing subepithelial PMN staining positive for membrane-associated APRIL and epithelium staining positive for secreted APRIL in CRS. Figure S4. Increased serum concentration of secreted APRIL in GPA when compared with HC and CRS. Figure S5. Immunohistochemistry showing expression of BCMA and D138, RANKL+ cells and TACI+ cells in CRS. [file ar4490-S1.doc]

**additional file - supporting information**

## Plasma cells within granulomatous inflammation display signs pointing towards autoreactivity and destruction in

## Granulomatosis With Polyangiitis

# Antje Mueller, ^1^ Christoph Brieske,^1^ Susanne Schinke,^1^ Elena Csernok,^1^ Wolfgang L. Gross,^1^ Katrin Hasselbacher,^2^ Jan Voswinkel,^3*^ Konstanze Holl-Ulrich^4*^

^1^Dept. of Rheumatology, University of Luebeck, Luebeck, Germany & Dept. of Rheumatology & Clinical Immunology, Medical Center Bad Bramstedt, Bad Bramstedt, Germany

^2^ Dept. of Otorhinolaryngology, University of Luebeck, Luebeck, Germany

^3^ Dept. of Hematology & Oncology, University Pierre et Marie Curie, Paris, France

^4^ Institute of Pathology, University of Luebeck, Luebeck, Germany

^*^ Both authors contributed equally.

corresponding author: Antje Mueller, PhD

Dept. of Rheumatology, University of Luebeck

Ratzeburger Allee 160, D-23538 Luebeck, Germany

phone: +49-(0)451-500-6565

fax: +49-(0)451-500-3650

email: antje.mueller@uksh.de

**Table 1.** Characteristics of patients at time of biopsy.

|  | **GPA (n=26)** | **CRS (n=20)** | **granulomatous disease control (n=2)** |
| --- | --- | --- | --- |
| age (median, range in years) | 52 (19-79) | 42 (15-80) | 45 (36-54) |
| sex | 15 males, 11 females | 13 males,  7 females | 1 male, 1 female |
| CRP  (mean±s.d., mg l^-1^) | 64.49±73.07 | n.d. | n.d. |
| ESR (mean±s.d., mm after the first hour) | 45.69 ±38.88 | n.d. | n.d. |
| Subgroup according to the classification by EULAR [1,2] | localised: 5,  early systemic: 6, generalised: 12,  refractory: 2 | n.a. | n.a. |
| BVAS v.3  (median, range) | 9 (3-27) | n.a. | n.a. |

All GPA patients fulfilled classification criteria according to European League Against Rheumatism recommendations [1,2]. Nasal biopsies were taken from patients with granulomatosis with polyangiitis (GPA), chronic rhinosinusitis (CRS) and sarcoidosis. There were no significant differences in age and gender between GPA and CRS (p>0.05). Signs for systemic inflammation (CRP, C-reactive protein; ESR, erythrocyte sedimentation rate) were heterogeneous. 15 GPA patients were untreated, 5 received cyclophosphamide plus steroids, 1 anti-TNF, 1 methotrexate plus steroids and 3 steroids only. The median cytoplasmic ANCA (cANCA) titer for GPA patients was 1:240. GPA disease activity was quantified using the Birmingham Vasculitis Activity Score (BVAS) v.3 [3]. Lung biopsies were taken from GPA (treatment and subgrouping were unknown for one patient) and RA patients. s.d. - standard deviation n.d. – not determined; n.a. – not applicable

**Table 2.** Detailed overview about characteristic histomorphological properties in nasal tissue (n=20) and lung (n=2) of GPA. “+” indicates presence, “-“ indicates absence, round brackets denote weak expression.

| **#** | **geographic necrosis** | **ill-defined**  **granuloma** | **scattered**  **giant cells** | **vasculitis** | **microabscess** | **bone**  **destruction** |
| --- | --- | --- | --- | --- | --- | --- |
| 1 | + | + | - | + | + | - |
| 2 | + | + | + | + | + | - |
| 3 | + | + | + | + | (+) | + |
| 4 | + | + | + | + | + | - |
| 5 | + | + | + | - | + | - |
| 6 | + | + | - | - | -. | - |
| 7 | + | + | + | - | + | - |
| 8 | + | + | (+) | + | +. | + |
| 9 | + | + | + | - | + | - |
| 10 | + | + | + | - | + | - |
| 11 | + | + | + | - | + | + |
| 12 | + | + | - | + | + | + |
| 13 | + | + | + | + | + | + |
| 14 | - | + | - | + | + | - |
| 15 | + | + | + | - | + | + |
| 16 | + | + | + | - | + | - |
| 17 | + | + | + | - | + | + |
| 18 | + | + | + | + | + | - |
| 19 | + | + | - | - | + | + |
| 20 | + | + | - | + | + | - |
| 21 | + | + | + | + | + | - |
| 22 | + | + | (+) | + | + | - |

**Table 3.** Characterisation of Ig V genes (n=41) derived from plasma cells (n=37) in nasal tissue in GPA (n=5). Couples of VL and VH chain genes are marked (bold).

| **#** | **germline gene** | **homology to  germline gene (%)** | **CDR**  **R** | **CDR**  **S** | **FR**  **R** | **FR**  **S** | **CDR3 length (aa)** |
| --- | --- | --- | --- | --- | --- | --- | --- |
| 87_36 | Vh1-18 | 91 | 3 | 0 | 12 | 5 | 15 |
| 87_35 | Vh2-5 | 100 | 0 | 0 | 0 | 0 | 10 |
| 87_10 | Vh3-23 | 88 | 0 | 3 | 15 | 11 | 14 |
| 87_04 | Vh3-30 | 94 | 5 | 2 | 0 | 1 | 18 |
| 87_15/1 | Vh3-53 | 86 | 5 | 3 | 14 | 4 | 12 |
| 87_21 | Vh4-4 | 94 | 0 | 0 | 10 | 1 | 10 |
| 87_29 | Vh4-61 | 95 | 2 | 1 | 4 | 3 | 14 |
| 87_15 | Vh5-51 | 92 | 4 | 3 | 7 | 2 | 15 |
| **88_42** | **Vh1-18** | **99** | **1** | **0** | **1** | **1** | **10** |
| **88_42** | **Vλ2-14** | **99** | **1** | **0** | **1** | **1** | **11** |
| **102_15** | **Vh1-3** | **88** | **4** | **3** | **13** | **12** | **19** |
| **102_15** | **Vk2-28** | **97** | **2** | **0** | **5** | **0** | **9** |
| 102_23 | Vh1-69 | 86 | 3 | 2 | 10 | 9 | 18 |
| 102_34 | Vh1-69 | 81 | 5 | 1 | 17 | 15 | 13 |
| 102_1 | Vh1-69 | 92 | 7 | 2 | 6 | 7 | 20 |
| 102_4 | Vh1-69 | 89 | 4 | 4 | 11 | 10 | 19 |
| **102_36** | **Vh3-30-3** | **90** | **4** | **1** | **14** | **5** | **14** |
| **102_36** | **Vk1-39** | **94** | **3** | **1** | **9** | **5** | **9** |
| 102_45 | Vh3-30-3 | 86 | 8 | 2 | 17 | 10 | 14 |
| 102_40 | Vh4-59 | 88 | 2 | 1 | 13 | 4 | 14 |
| 102_44 | Vh4-59 | 100 | 0 | 0 | 1 | 0 | 13 |
| 255_28 | Vh1-2 | 93 | 5 | 0 | 8 | 5 | 21 |
| 255_2 | Vh1-46 | 98 | 3 | 0 | 2 | 0 | 15 |
| 255_6 | Vh1-69 | 98 | 0 | 0 | 4 | 1 | 20 |
| 255_26 | Vh1-69 | 96 | 3 | 1 | 7 | 1 | 21 |
| **255_42** | **Vh1-69** | **98** | **2** | **1** | **3** | **0** | **24** |
| **255_42** | **Vk1-39** | **98** | **3** | **0** | **2** | **1** | **9** |
| 255_41 | Vh3-49 | 98 | 1 | 0 | 3 | 0 | 16 |
| 255_38 | Vh4-30-2 | 91 | 7 | 2 | 9 | 5 | 12 |
| 280_10 | Vh1-2 | 93 | 4 | 0 | 11 | 3 | 19 |
| 280_16 | Vh1-69 | 93 | 8 | 1 | 5 | 5 | 21 |
| 280_30 | Vh1-69 | 92 | 6 | 5 | 5 | 5 | 26 |
| 280_35 | Vh1-69 | 89 | 9 | 1 | 12 | 8 | 18 |
| 280_42/1 | Vh1-69 | 93 | 10 | 2 | 7 | 1 | 18 |
| 280_14 | Vh2-5 | 99 | 0 | 0 | 3 | 1 | 7 |
| 280_25 | Vh3-74 | 93 | 6 | 0 | 6 | 5 | 14 |
| 280_36 | Vh3-74 | 93 | 7 | 2 | 3 | 4 | 15 |
| 280_17 | Vh4-31 | 85 | 8 | 1 | 16 | 8 | 15 |
| 280_28 | Vh4-39 | 95 | 6 | 1 | 4 | 0 | 20 |
| 280_42 | Vh4-4 | 88 | 4 | 4 | 8 | 10 | 15 |
| 280_19 | Vh-5-51 | 93 | 6 | 0 | 6 | 6 | 13 |

**Table 4.** Summary of histomorphological features characterising ectopic lymphoid structures (ELS) in GPA (nasal tissue, lung) compared to unspecific CRS.

| histomorphological features | GPA **(n / n)** | CRS **(n / n)** |
| --- | --- | --- |
| CD3^+^ T cells in follicular distribution | 21 / 25 | 17 / 20 |
| CD20^+^ B cells in follicular distribution | 21 / 24 | 18 / 20 |
| CD21^+^ cellular network | 11 / 20 | 14 / 20 |
| CD23^+^ cellular network | 16 / 25 | 17 / 20 |
| CD35^+^ cellular network | 15/ 25 | 12 / 20 |
| PNAd^+^ HEV-like structure | 11 / 18 | 8 / 11 |
| CXCL13^+^ cells in follicular structures | 13 / 20 | 9 / 20 |
| CCL21^+^ vascular structure | 13 / 20 | 4 / 20 |
| CD4^+^ cell aggregates | 13 / 16 | 17 / 19 |
| CD8^+^ cell aggregates | 13 / 20 | 12 / 17 |
| CD57^+^ cells in follicular structures | 12 / 15 | 16 / 18 |

**Table 5.** Summary of APRIL expression by characteristic histomorphological features in nasal tissue in GPA.

| **phenotypical features** | **microabscess  (n / n)** | **multinucleated giant cells & macrophages in granuloma**  **(n / n)** |
| --- | --- | --- |
| proteinase 3^+^ neutrophil granulocytes^a^  CD68^+^ macrophages & mutinucleated giant cells  produced APRIL^+^  secreted APRIL^+^ | 16 / 16  0 / 16  13 / 13  0 / 13 | 0 / 13  16 / 16  13 / 13  13 / 13 |

^a^ Proteinase 3 (PR3) was detected using immunohistochemistry with anti-PR3 antibody (WGM2).

**Figure 1** Sequence analysis of plasma-cell derived Ig VH genes from nasal mucosa in GPA showing the (A) number of somatic mutations, (B) VH gene repertoire and (C) length (number of amino acids; aa) of the CDR3. The light and medium grey bars indicate somatic mutations, VH family and CDR3 length of B cells taken from GPA and healthy controls (HC) [4-6]. None of the differences reached statistical significance (p<0.05), except for the CDR3 length, which was prolonged in GPA when compared to peripheral blood B lymphocytes in HC [5] (in terms of aa numbers but not relative abundance; Mann Whitney test: p<0.02).

A

B

C

**Figure 2** Expression of homeostatic lymphoid chemokines CXCL13 and CCL21. Immunohistochemistry depicting CXCL13^+^ cells (red color) within follicular structures in GPA (A) and CRS (B) and vascular CCL21^+^ cells (red color, arrows) in GPA (C) and CRS (D).


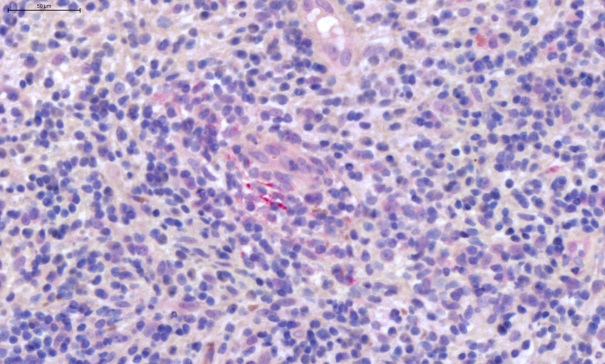

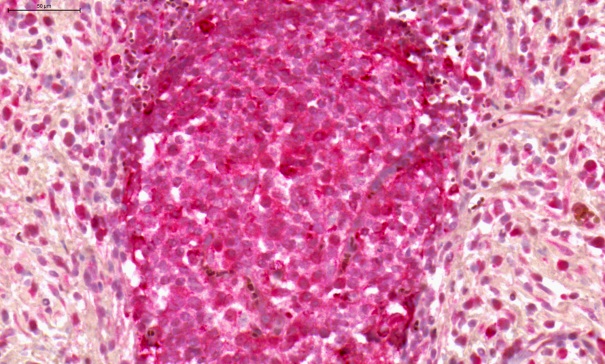

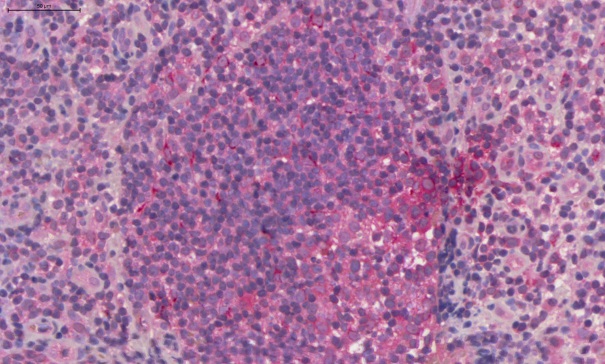


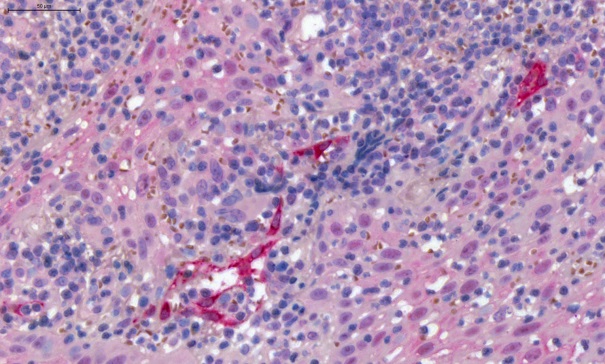


**A**

**B**

**C**

**D**

**Figure 3** Immunohistochemistry showing subepithelial PMNs staining positive for membrane-associated APRIL (A: brown color, arrows) and epithelium staining positive for secreted APRIL (B: brown color, arrow) in CRS.


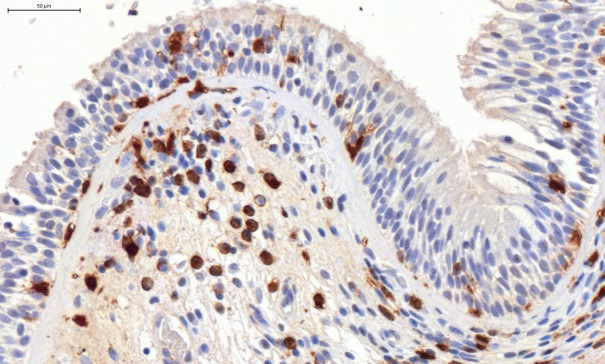

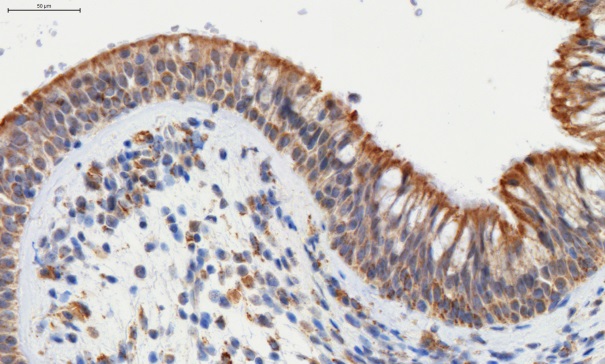


**A**

**B**

**Figure 4** Increased serum concentration of secreted APRIL in GPA (black circles) when compared to HC (white circles) and CRS (white triangles). The mean values±SEM are shown. *** p<0.001. There was no correlation with GPA disease activity. Patient characteristics: m/f: HC 5/8; CRS 5/5; GPA 10/10, age: HC 38.62±2.95; CRS 41.2±6.4; GPA 49.1±4.02 (p>0.05), BVAS v.3 median 3; range 0-15, cANCA/PR3-ANCA^+^ n=15; cANCA/PR3-ANCA^-^ n=5.

**Figure 5** Immunohistochemistry showing expression of BCMA & CD138 (A: BCMA: brown color; CD138: red color, arrow; n=5), RANKL^+^ cells (B: brown color, arrows; n=10) and TACI^+^ cells (C: brown color, arrow; n=10) in CRS.


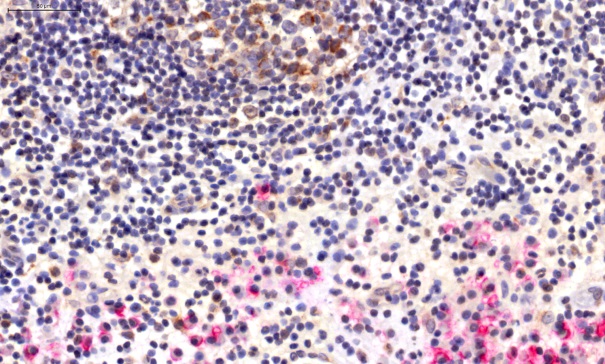
 **
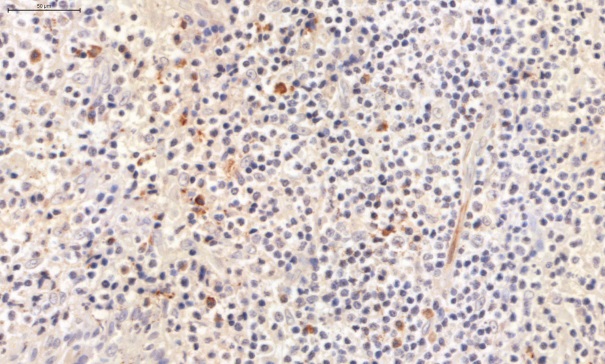
**

**A**

**B**

**
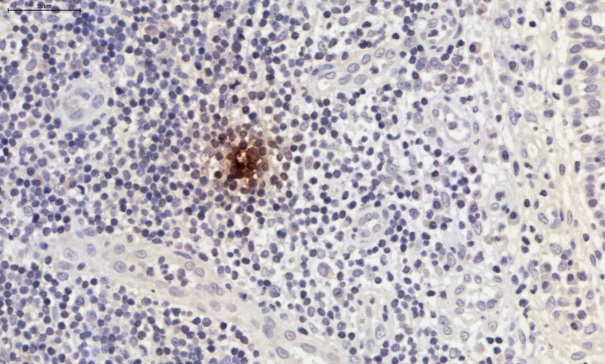
**

**C**

**References**

[1] Hellmich B, Flossmann O, Gross WL, Bacon P, Cohen-Tervaert JW, Guillevin L, Jayne D, Mahr A, Merkel PA, Raspe H, Scott DGI, Witter J, Yazici H, Luqmani RA, on behalf of the European Vasculitis study group: **EULAR recommendations for conducting clinical studies and/or clinical trials in systemic vasculitis: focus on anti-neutrophil cytoplasm antibody-associated vasculitis.** *Ann Rheum Dis* 2007, **66**:605-617.

[2] Watts R, Lane S, Hanslik T, Hauser T, Hellmich B, Koldingsnes W, Mahr A, Segelmark M, Cohen-Tervaert JW, Scott D: **Development and validation of a consensus methodology for the classification of the ANCA-associated vasculitides and polyarteritis nodosa for epidemiological studies.** *Ann Rheum Dis* 2007, **66**:222-227.

[3] Mukthyar C, Lee R, Brown D, Carruthers D, Dasgupta B, Dubey S, Flossmann O, Hall C, Hollywood J, Jayne D, Jones R, Lanyon P, Muir A, Scott D, Young L, Luqmani R: **Modification and validation of the Birmingham vasculitis activity score (version 3).** *Ann Rheum Dis* 2009, **68**:1827-1832.

[4] Voswinkel J, Assmann G, Held G, Pitann S, Gross WL, Holl-Ulrich K, Herlyn K, Mueller A: **Single cell analysis of B lymphocytes from Wegener’s granulomatosis: B cell receptors display affinity maturation within the granulomatous lesions.** *Clin Exp Immunol* 2008, **154**:339-345.

[5] Brezinschek HP, Brezinschek RI, Lipsky PE: **Analysis of the heavy chain repertoire of human peripheral B cells using single-cell polymerase chain reaction.** *J Immunol* 1995, **155**:190-202.

[6] Boursier L, Dunn-Walters DK, Spencer J: **Characteristics of Ig V_H_ genes used by human intestinal plasma cells from childhood.** *Immunology* 1999, **97**:558-564.
